# Clinical and cellular features in patients with primary autosomal recessive microcephaly and a novel *CDK5RAP2* mutation

**DOI:** 10.1186/1750-1172-8-59

**Published:** 2013-04-15

**Authors:** Lina Issa, Katrin Mueller, Katja Seufert, Nadine Kraemer, Henning Rosenkotter, Olaf Ninnemann, Michael Buob, Angela M Kaindl, Deborah J Morris-Rosendahl

**Affiliations:** 1 Institute of Cell Biology and Neurobiology, Charité University Medicine, Berlin, Germany; 2 Department of Pediatric Neurology, Charité University Medicine, Augustenburger Platz 1, Berlin 13353, Germany; 3 Institute of Human Genetics, Albert-Ludwigs University Medical Center Freiburg, Breisacherstr 33, Freiburg 79106, Germany; 4 Sozialpaediatrisches Zentrum, Klinikum, Ludwigsburg, Germany; 5 Current Address: National Heart and Lung Institute, Imperial College London, London, UK

**Keywords:** Microcephaly, CDK5RAP2 mutation, Cell division, CDK5RAP2 protein

## Abstract

**Background:**

Primary autosomal recessive microcephaly (MCPH) is a rare neurodevelopmental disorder that results in severe microcephaly at birth with pronounced reduction in brain volume, particularly of the neocortex, simplified cortical gyration and intellectual disability. Homozygous mutations in the Cyclin-dependent kinase 5 regulatory subunit-associated protein 2 gene *CDK5RAP2* are the cause of MCPH3. Despite considerable interest in MCPH as a model disorder for brain development, the underlying pathomechanism has not been definitively established and only four pedigrees with three *CDK5RAP2* mutations have been reported. Specifically for MCPH3, no detailed radiological or histological descriptions exist.

**Methods/Results:**

We sought to characterize the clinical and radiological features and pathological cellular processes that contribute to the human MCPH3 phenotype. Haplotype analysis using microsatellite markers around the MCPH1-7 and *PNKP* loci in an Italian family with two sons with primary microcephaly, revealed possible linkage to the MCPH3 locus. Sequencing of the coding exons and exon/intron splice junctions of the *CDK5RAP2* gene identified homozygosity for the novel nonsense mutation, c.4441C > T (p.Arg1481*), in both affected sons. cMRI showed microcephaly, simplified gyral pattern and hypogenesis of the corpus callosum. The cellular phenotype was assessed in EBV-transformed lymphocyte cell lines established from the two affected sons and compared with healthy male controls. CDK5RAP2 protein levels were below detection level in immortalized lymphocytes from the patients. Moreover, mitotic spindle defects and disrupted γ-tubulin localization to the centrosome were apparent.

**Conclusion:**

These results suggest that spindle defects and a disruption of centrosome integrity play an important role in the development of microcephaly in MCPH3.

## Introduction

Primary autosomal recessive microcephaly (MCPH) delineates a genetically heterogeneous and rare subgroup of congenital microcephalies characterized by a pronounced reduction of brain volume, particularly of the neocortex, simplified gyral pattern and intellectual disability [1,2]. Homozygous mutations of the Cyclin-dependent kinase 5 regulatory subunit-associated protein 2 gene, *CDK5RAP2* (OMIM*608201), were identified in 2005 as a cause for MCPH type 3 (MCPH3, OMIM#604804) [3]. To date, three different mutations have been identified: two in three Pakistani families and one mutation in a Somali patient: (i) a nonsense mutation in exon 4 (c.246T > A, p.Y82X), (ii) an A to G transition in intron 26 (c.4005-15A > G, p.R1334SfsX5) introducing a new splice acceptor site, a frame shift and a premature stop codon, and (iii) a nonsense mutation in exon 8 (c.700G > T, p.E234X) [3-5]. All three mutations have been proposed, but not shown, to lead to a truncated protein and a loss of CDK5RAP2 function.

CDK5RAP2 is associated with the centrosome, microtubuli and Golgi apparatus, is enriched in neural progenitors within the ventricular and subventricular zone of the immature brain, can be also detected in glial cells and early neurons, and is strongly downregulated with brain maturation [6,7]. One current model for the microcephaly phenotype caused by *CDK5RAP2* mutation invokes a premature shift from symmetric to asymmetric neural progenitor cell divisions with a subsequent depletion of the progenitor pool and a reduction in the final number of neurons, and decreased cell survival [6,8]. Underlying mechanisms include a deregulation of the role of CDK5RAP2 in centrosome function, spindle assembly and/or response to DNA damage [6,8]. Despite considerable interest in MCPH as a neural stem cell defect and window into the control of neurogenesis in humans, the underlying pathomechanisms have not been definitively established and specifically for MCPH3, no detailed radiological descriptions of patients or functional analyses in patient samples have been reported to date.

In the present study, we report a novel *CDK5RAP2* mutation and describe for the first time in detail the clinical, radiological and cellular phenotype in two MCPH3 patients of European descent. We are thereby able to attribute the microcephaly phenotype in MCPH3 at least partially to a mitotic spindle defect and centrosome disorganization.

## Material and methods

### Patients

Informed consent was obtained from the parents of the patients for the molecular genetic analysis, the publication of clinical data, magnetic resonance images (MRI) and studies on immortalized lymphocytes (LCLs). DNA was extracted from EDTA blood samples using the Illustra BACC2 DNA extraction kit (GE Healthcare, Munich, Germany). Samples from microcephaly patients and controls were used in this study with approval from the local ethics committees of the Charité and the Freiburg University (approval nos. EA1/212/08 and 494/11, respectively).

### Haplotype analysis using microsatellite markers

Six microsatellite markers were selected for each of the MCPH1 to 7 and *PNKP* loci, so that three markers were located on each side of each gene. The markers flanking the *CDK5RAP*2 gene were: CHLC.GGAA23B10, D9S258, D9S2152, D9S103, D9S116 and D9S1823. PCR was performed with 1 ng patient DNA and primer pairs in which the forward primer was always labeled with 6-FAM. PCR fragments were resolved by capillary electrophoresis on an ABI 3100 sequencer. Fragment analysis was performed using GeneScan software (Applied Biosystems, Foster City). Haplotypes were constructed in the family by inspection of the microsatellite fragment lengths.

### PCR and DNA sequencing

Thirty-eight coding exons of the *CDK5RPAP2* gene and at least 50 bp of the intronic, exon-flanking sequence were analyzed by PCR (Taq Polymerase, Qiagen, Hilden, Germany), and cycle sequencing using the ABI Prism BigDye Terminator Cycle Sequencing Ready Reaction Kit Version 1.1 (Applied Biosystems, Darmstadt, Germany). Capillary electrophoresis was performed using an ABI 3100 sequencer (Applied Biosystems, Foster City, CA, USA). Sequence data were analyzed using SeqPilot DNA sequence analysis software (JSI, Kippenheim, Germany). The database sequence NM_018249 for the *CDK5RAP2* gene was used as reference, and primers were developed in our laboratory (available on request).

### Establishment of Ebstein-Barr virus-transformed lymphocytes and culture

Ebstein-Barr virus-transformed lymphocytes (LCLs) were established according to the protocol published by Neitzel et al. 1986 [9]. Non-adherent LCLs were cultured in RPMI 1640 with L-Glutamine (Invitrogen, Darmstadt, Germany) supplemented with 20% v/v fetal bovine serum (Invitrogen) and 1% v/v penicillin-streptomycin (Sigma-Aldrich, Taufkirchen, Germany).

### Immunocytology

For fixation, cells were plated on Poly-L-lysine (Sigma-Aldrich) coated coverslips, cultured for 30 min in standard conditions, and incubated in 37°C PFA 4% for 10 min prior to rinsing with phosphate buffered saline (PBS 1×). Coverslips were further incubated at room temperature (RT) in staining buffer (0,2% gelatin, 0,25% Triton X-100 in PBS 1×) for 20 min and subsequently in 10% donkey normal serum (DNS) in staining buffer for 30 min for blocking. Coverslips were incubated overnight at 4°C with primary antibodies in the staining buffer containing 10% DNS followed by an incubation with the corresponding secondary antibodies for 2 h at RT. Nuclei were labeled with 4’,6-diamidino-2-phenylindole (DAPI, 1:1000, Sigma-Aldrich). Fluorescently labeled cells were analyzed and imaged by a fluorescent Olympus BX51 microscope with the software Magnafire 2.1B (2001) (Olympus, Hamburg, Germany), and all images were processed using Adobe Photoshop. The anti-CDK5RAP2 antibody (HPA035820; 1:200) utilized in this study recognizes amino acids 1307–1390 of CDK5RAP2 which is unique for the human CDK5RAP2 protein sequence (accession no. NP_060719.4, Uniprot Q96SN8). Further primary antibodies were as follows: mouse anti-γ-tubulin (T5326, Sigma-Aldrich, 1:5000), mouse anti-alpha-tubulin (T9026, Sigma-Aldrich; 1:1500), mouse anti-pericentrin (ab28144, Abcam; 1:1000), mouse anti-acetylated alpha-tubulin (T6793, Sigma-Aldrich; 1:1000), mouse anti-GM130 (610823, BD Biosciences; 1:1000).

### Protein extraction procedure and Western blot

Protein extracts for Western blots were isolated from LCLs by homogenization in radio-immunoprecipitation assay (RIPA) buffer containing 1 mM phenylmethylsulfonyl fluoride (PMSF; Sigma-Aldrich) and 1 protease inhibitor cocktail tablet per 10 ml RIPA buffer (Complete Mini; Roche Diagnostics, Mannheim, Germany), 20 min incubation on ice and centrifugation at 4°C for 10 min at 3000 g and for 20 min at 16000 g. Protein concentrations were determined using a bicinchoninic acid (BCA) based assay, according to the instructions of the manufacturer (BCA Protein Assay Kit; Pierce Biotechnology, Rockford, IL, USA). Protein extracts (30 μg per sample) were denaturated in Laemmli sample loading buffer at 95°C for 5 min, separated by sodium dodecyl sulphate polyacrylamide gel electrophoresis (SDS-PAGE) and electrophoretically transferred in transfer buffer in a semi-dry fashion using Trans-Blot SD Semi-Dry transfer cell (Bio-Rad, Munich, Germany) onto nitrocellulose membrane (Bio-Rad, Munich, Germany). The membranes were incubated for 1 h at RT in blocking buffer (TBS-T 1x with 5% bovine serum albumin (BSA)), rinsed three times with TBS-T (1x) for 8 min each at RT on a shaker and then incubated overnight at 4°C with rabbit anti-CDK5RAP2 (1:200, HPA035820, Sigma-Aldrich; also verified with antibody from Abnova PAB17507, 1:200), mouse anti-γ-tubulin (1:5,000) or mouse anti-CHK1 (1:1000, Sigma-Aldrich) antibodies. After incubation with the corresponding secondary antibodies donkey anti-rabbit (1:2000; Amersham Biosciences, Freiburg, Germany) and goat anti-mouse (1:10,000; Dako, Hamburg, Germany), the immunoreactive proteins were visualized using a technique based on a chemiluminescent reaction. The gel pictures were obtained with a Bio-Rad imager (Bio-Rad laboratories, Munich, Germany). Western blot experiments were run in triplicate.

## Results

### Phenotype of patients with MCPH3

**Figure 1:**
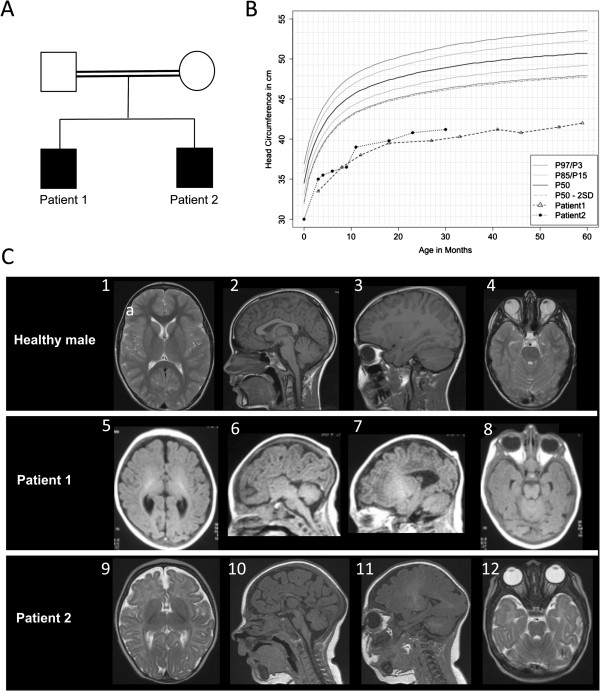
**Clinical features of MCPH3 patients with novel *CDK5RAP2 *gene mutation.** (**A**) Pedigree of the Italian family: the parents were third degree cousins. (**B**) Development of the OFC of both patients, from age 2 months to 5 years in patient 1 (triangles) and birth to 3 years 10 months in patient 2 (dots) (centiles refer to WHO Child Growth Standards) [10,11]. The OFC of patient 2 was below −3.5 SD at birth and further decreased to about −6.4 SD by the age of 3 years and 10 months. (**C**) T1/2-weighted magnetic resonance images (MRI) of patient 1 at age 2,5 months (5–8) and patient 2 at 3 months (9–12) compared to those of a healthy 3-year old boy. The reduced size of the brain with extra-axial spaces (5, 7, 9–11), sloping forehead (6, 7, 11), simplified gyral pattern frontally with shallow, wide sulci (5, 7, 9) and corpus callosum agenesis (6) and hypogenesis (10) are apparent.

The first son (Patient 1) was born prematurely to Italian parents who were third cousins (Figure 1A), at gestational week 35, with a birth weight of 2570 g (exact birth parameters not available). At the age of 3 months, he weighed 4530 g (370 g < 3^rd^ centile), was 53 cm long (3,9 cm <3^rd^ centile, -4.1 SD), and had an occipital-frontal head circumference (OFC) of 33,5 cm (4,8 cm < 3^rd^ centile, -5.9 SD). Further progression of the OFC is shown in Figure 1B. A closed fontanel, a simian crease, an abdominal hernia and slightly increased muscle reflexes, but no pyramidal signs were observed. Skeletal scintigraphy ruled out craniosynostosis (premature closure of the fontanels) as a cause of microcephaly. The results of routine blood tests including a full blood count, electrolytes, liver, kidney and thyroid parameters, CK and tests for TORCH and metabolic diseases were normal. Clearly defined areas of hyperpigmentation were apparent on the medial side of the right leg, left ankle and left pectoral. Chromosome analysis revealed a normal male karyotype. The results of an ophthalmological investigation as well as ultrasound of the kidneys and hip joints were normal. At age 4,5 months, audio-acoustic emissions could not be detected, and brainstem audiometry revealed a slightly elevated absolute threshold of hearing of 35–40 dB. However at age 1 year tests (BERA) were repeated, and his hearing was found to be normal. On cranial MRI, microencephaly, simplified gyral pattern, particularly frontally, and agenesis of the corpus callosum were apparent (Figure 1C, 5–8). On EEG, the oscillations were slower than expected for age, but epileptic discharges were absent. His initial short stature appeared to become less prominent with age, so that at age 9 years his height was average. At age 8 years the following tests were performed, all with normal results: full blood count, differential blood analysis, glucose, creatinine, CK, LDH, GOT, GPT, TSH, T_4_. An IQ test revealed intellectual disability (IQ 50–69) with slight developmental delay in speech and motor functions, and a short concentration span. According to the Munich Functional Development test (MFE II) at age 5 years his speech and understanding were at an age of 27–36 months and expressive speech was at an age of 25–34 months. Moreover the boy suffered from a tic disorder manifesting as repetitive blinking, nodding or smacking of the lips. He had behavioral problems, with hyperactivity, bouts of rage and aggression, which were severe enough to necessitate short-term admission to a childrens’ psychiatric hospital at age 11 years. He was socially inept, easily upset and irascible. However, the behavioral problems responded well to treatment with risperidone.

**Figure 2:**
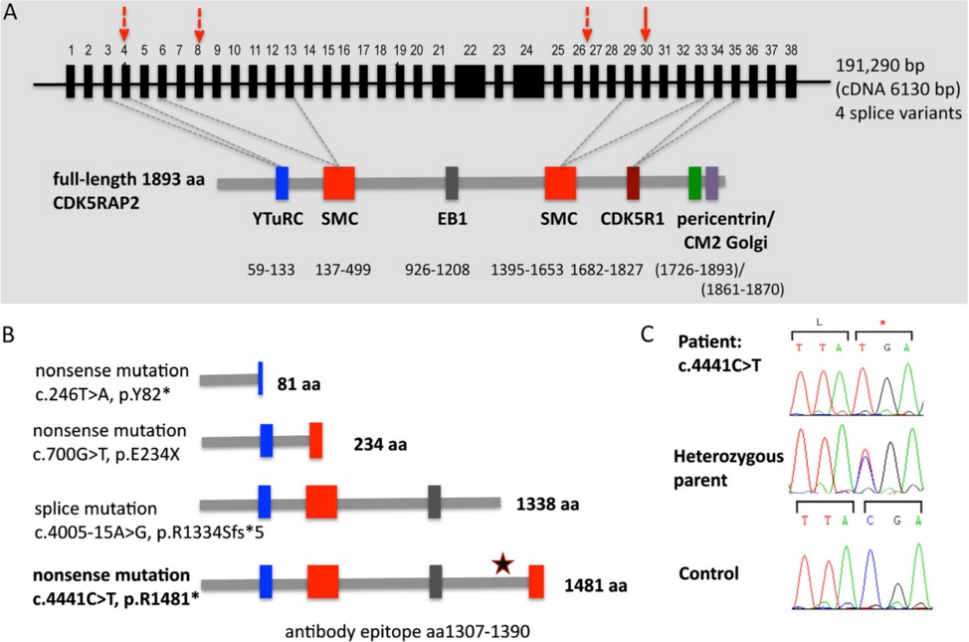
**Novel *CDK5RAP2 *gene mutation in patients with MCPH3.** (**A**) Schematic representation of the domain structure of CDK5RAP2 showing the positions and the effects of *CDK5RAP2* mutations that cause MCPH3. Nonsense mutation c.4441C > T is shown as a solid arrow and previously described *CDK5RAP2* mutations as dotted arrows. (**B**) Predicted protein products that result from the *CDK5RAP2* gene mutations. The star indicates the binding site of the anti-CDK5RAP2 antibody utilized in this study. (**C**) Electropherogram shows mutation c.4441C > T in patient 1 and the normal sequence in a control.

The second son (Patient 2) was born in the 40^th^ week of gestation with a birth weight of 3130 g (25^th^-50^th^ centile), 49 cm long (25^th^-50^th^ centile) and OFC of 30 cm (2,1 cm <3^rd^ centile, -3.5 SD). Apgar scores were 9/10/10. Full blood count, differential blood analysis, TORCH and newborn metabolic screening, CK, electrolytes, liver and kidney parameters were all normal. Shortly after birth, intermittent breathing pauses were observed, but never again observed thereafter. He had dysmorphic features including a sloping forehead, low set ears, a relatively high-arched palate and simian creases. Similar to his brother, he had relatively large, map-like areas of hyperpigmentation with well-defined borders: four on his inner right leg, five around the ankle, and one on his left pectoral. The further development of the OFC is shown in Figure 1B. Chromosome analysis and tests for lactate, LDH, GOT, GPT and AP at age six months were normal. All further investigations including electrocardiogram (ECG), ultrasound of the cranium, hips, kidneys, adrenals, bladder as well as an ophthalmological examination yielded normal results. Cranial MRI at the age of five months showed microencephaly, simplified gyral pattern particularly of the frontal lobes and corpus callosum hypogenesis (Figure 2C, 9–12). Moreover, an increased space in the posterior fossa, consistent with a megacisterna magna most probably secondary to mild cerebellar hypoplasia, could be visualized (Figure 2C, 10). At age 11 months, the patient had developed normally with respect to motor skills; however, two years later mild motor and intellectual developmental delay was noted with an index value of 56 for cognitive development on the Bayley Scales of Infant Development. Although he had relatively good speech development, especially considering that he was brought up to be bilingual, he had similar behavioral problems to his brother, with temper tantrums, problems with motivation and concentration and was not able to attend a regular nursery school. Neither of the patients had seizures by ages 11 years (Patient 1) and 6 years (Patient 2). The patients’ parents did not consent to the publication of photos of the patients. The clinical findings in both patients as well as those of previously published patients are summarized in Table 1.

**Table 1:** Summary of the clinical features in the patients described in this report (P1 = patient 1, P2 = patient 2), together with those from previous publications (− = not reported)

***CDK5RAP2 *mutation**	**Exon 4: 246 T > A**	**Exon 4: 246 T > A**	**Exon 8: 700G > T**	**Intron 26: IVS26-15A > G**	**Exon 30: 4441C > T**
**No. (sex) patients, ethnicity**	4 (3f, 1 m) Northern Pakistani	4 (2f, 2 m) Northern Pakistani	1 (f) Somalian	2 (f) Northern Pakistani	2 (m) Italian
**Microcephaly at birth**	yes	yes	yes (−3.7 SDS)	yes	yes (−3.5 SDS)
**Microcephaly later**	−6 to −8 SDS	−4 to −7 SDS	−8.9 SDS	−5 to −7 SDS	−3.5 to −5.9 SDS
**Height/weight at birth**	-	1 patient with low birth weight (1.9 kg at term)	low birth weight at term (2.4 kg, -2.4 SDS) and length (−3.7 SDS)	-	P1: height −4.1 SD at 3 m; P2: Normal (25^th^-50^th^ centile)
**Height/weight later**	-	-	-	-	P1: normal at age 9y; P2: 3^rd^ centile at age 2y
**Intellectual disability**	mild-moderate	mild-moderate (IQ51-65)	-	moderate	mild-moderate (IQ50-69)
**Speech disorder**	-	-	-	-	yes
**Sloping forehead**	yes	yes	yes	yes	yes
**Other malformations**	-	-	-	-	Simian crease, large map-like hyperpigmentation
**Milestones**	-	-	slightly delayed	-	slightly delayed
**Muscular Hypotonia**	-	-	yes	-	high-arched palate in patient 2
**Muscle reflexes**	-	-	-	-	increased in patient 1 (no pyramidal signs)
**Joint laxity**	-	-	yes	-	no
**Decreased muscle bulk**	-	-	yes	-	no
**Behavioral problems**	-	-	no	-	yes, severe irritability and aggression
**Hearing**	one patient (f) with profound congenital sensorineural deafness	-	postnatal onset moderate-severe sensorineural deafness	-	no
**Malignancy**	acute lymphoblastic leukemiain one female patient	-	-	-	no
**Seizures**	one female patient	-	-	-	no
**cMRI**	-	-	microcephaly, no structural abnormality	not reported	microcephaly, simplified gyral pattern frontally, corpus callosum agenesis/hypogenesis (P1 and P2)
**Other**			gastrostomy feeding needed		abdominal hernia (P1)
**Reference**	[3]	[4]	[5]	[3]	this study

### A novel *CDK5RAP2* mutation in a family with MCPH

Haplotype analysis using microsatellite markers revealed that both affected sons in the family were homozygous for a haplotype surrounding the MCPH3 locus, shared by the heterozygous parents, who are third degree cousins. Possible linkage consistent with compound heterozygosity in both sons was also suggested for the *MCPH4, MCPH7* and *PNKP* loci; however, sequencing of the *STIL* and *PNKP* genes did not reveal mutations. Sequencing of *CDK5RAP2* showed that both affected sons were homozygous for the mutation c.4441C > T, which results in the nonsense mutation p.Arg1481*; both parents were heterozygous for the mutation (Figure 2). The resulting CDK5RAP2 protein is predicted to result in a truncation that affects the second SMC, the pericentrin, and the Golgi binding sites (Figure 2).

### Cellular phenotype of patients with *CDK5RAP2* gene mutation

**Figure 3:**
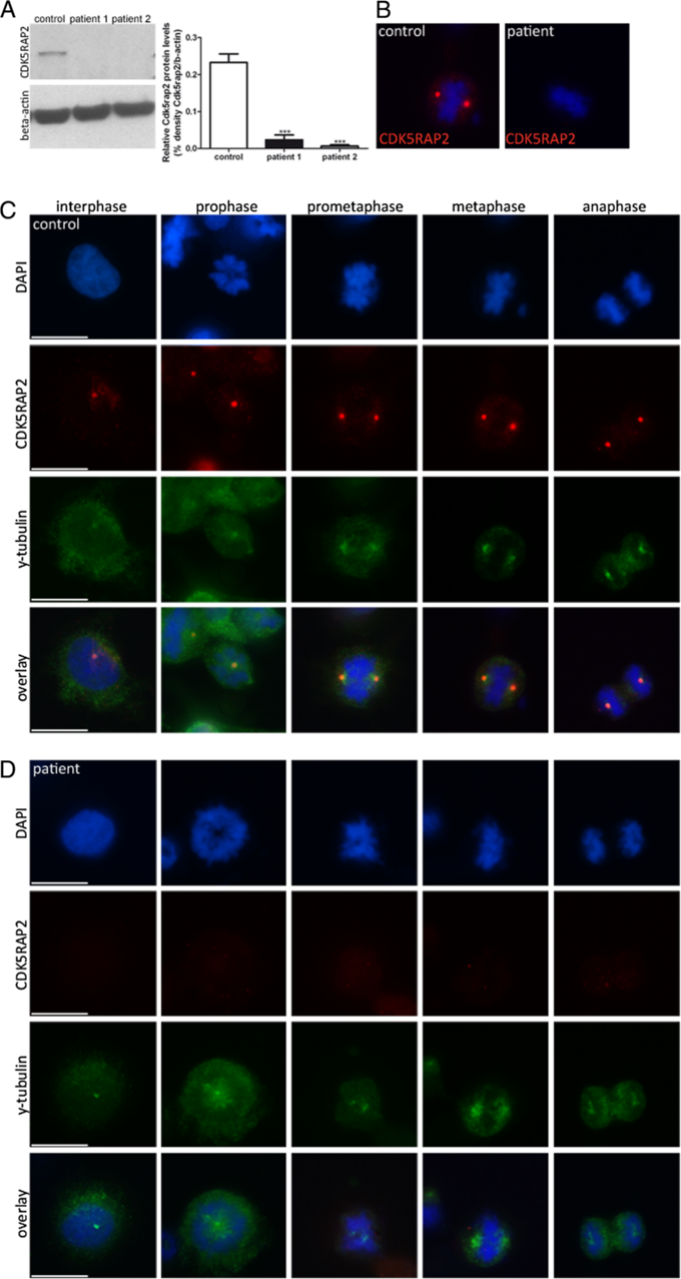
**CDK5RAP2 in immortalized lymphocytes and dispersion of centrosomal protein γ-tubulin in *CDK5RAP2 *mutant patients cells.** CDK5RAP2 protein levels were below detection level in immortalized lymphocytes from both patients with the c.4441C > T *CDK5RAP2* mutation when assessed by (**A**) Western blot and (**B**) immunocytology. Subcellular localization of CDK5RAP2 and γ-tubulin throughout the cell cycle in immortalized lymphocytes of (**C**) controls and (**D**) MCPH3 patient 2. In controls, centrosomal CDK5RAP2 levels were weak during interphase, increased in the subsequent prophase and remained high throughout mitosis until telophase, when signals dropped to interphase levels. In the patient cells the alignment of the chromosomes at the spindle poles was less precise than in the control cells. Cells were stained with CDK5RAP2 (red), γ-tubulin (green) as a centrosomal marker, and DNA is stained with DAPI (blue). Scale bars 10 μm. Western blot results reveal that total γ-tubulin protein levels are similar in patients and controls (Additional file 1: Figure S1).

**Figure 4:**
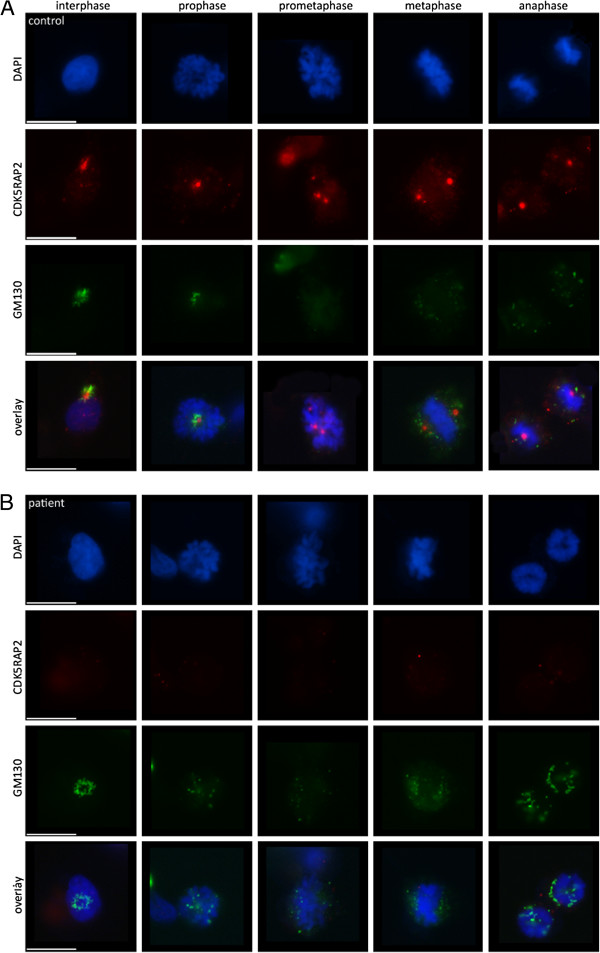
**Golgi apparatus marker GM130 in *CDK5RAP2 *mutant patients cells.** Subcellular localization of CDK5RAP2 and GM130 throughout the cell cycle in immortalized lymphocytes of (**A**) control and (**B**) MCPH3 patient 2. Golgi fragmentation appeared to occur earlier during mitosis,already showing a dispersed signal during prophase in patient cells.

We investigated the pathogenicity of the identified missense mutation in immortalized lymphocytes (LCLs) from the two patients with MCPH3 and from controls. In control LCLs, CDK5RAP2 localized to the centrosomes during each stage of the cell cycle (Figure 3). Consistent with studies in murine cells [12], centrosomal CDK5RAP2 levels were relatively low during interphase, increased in the subsequent prophase and remained high throughout mitosis until telophase, when signals dropped to interphase levels. CDK5RAP2 further accumulates at the Golgi apparatus [13], and we detected a partial colocalization with the cis-Golgi matrix protein GM130 in LCLs during inter- and prophase. In prometaphase, the Golgi apparatus begins to fragment and loses its pericentriolar location close to CDK5RAP2 (Figure 4). In metaphase and anaphase the fragments were still somewhat dispersed in the cytoplasm but some could already be detected in the proximity of the CDK5RAP2-positive centrosomes. During telophase, cytokinesis separates the two daughter cells, and reassembly of the Golgi apparatus occurs in the centrosomal region of each daughter cell. In *CDK5RAP2* mutant LCLs, CDK5RAP2 levels were below detection levels when assessed through immunocytology and western blots using two antibodies that bind to different positions at the C-termini of full-length CDK5RAP2 (Figure 3). Since the Golgi domain described previously at the C-terminus [13] is predicted to be lost in our patients, we further analyzed the Golgi integrity through immunostaining with GM130. GM130 immuno-signal clusters were apparent in interphase cells from patients. However, Golgi fragmentation appeared to occur earlier during mitosis and had disappeared by prophase (Figure 4).

**Figure 5:**
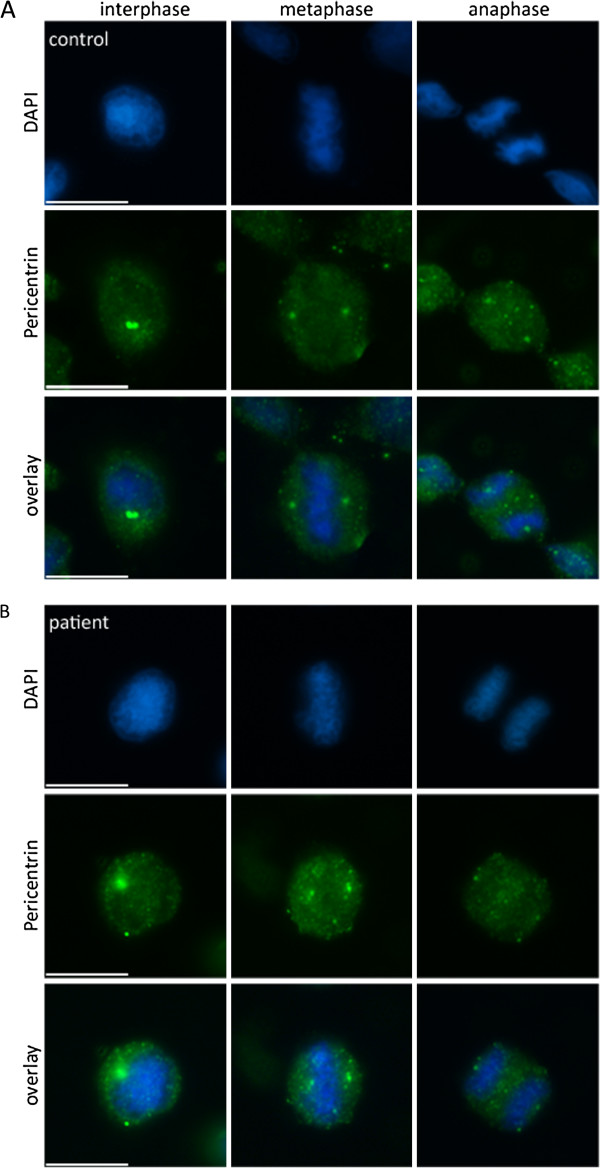
**Pericentrin in *CDK5RAP2 *mutant patient and control cells.** No difference was detected regarding the subcellular localization of pericentrin throughout the cell cycle in immortalized lymphocytes of (**A**) control and (**B**) MCPH3 patients (here patient 2), however the staining in patients was weaker and the signal more diffuse.

**Figure 6:**
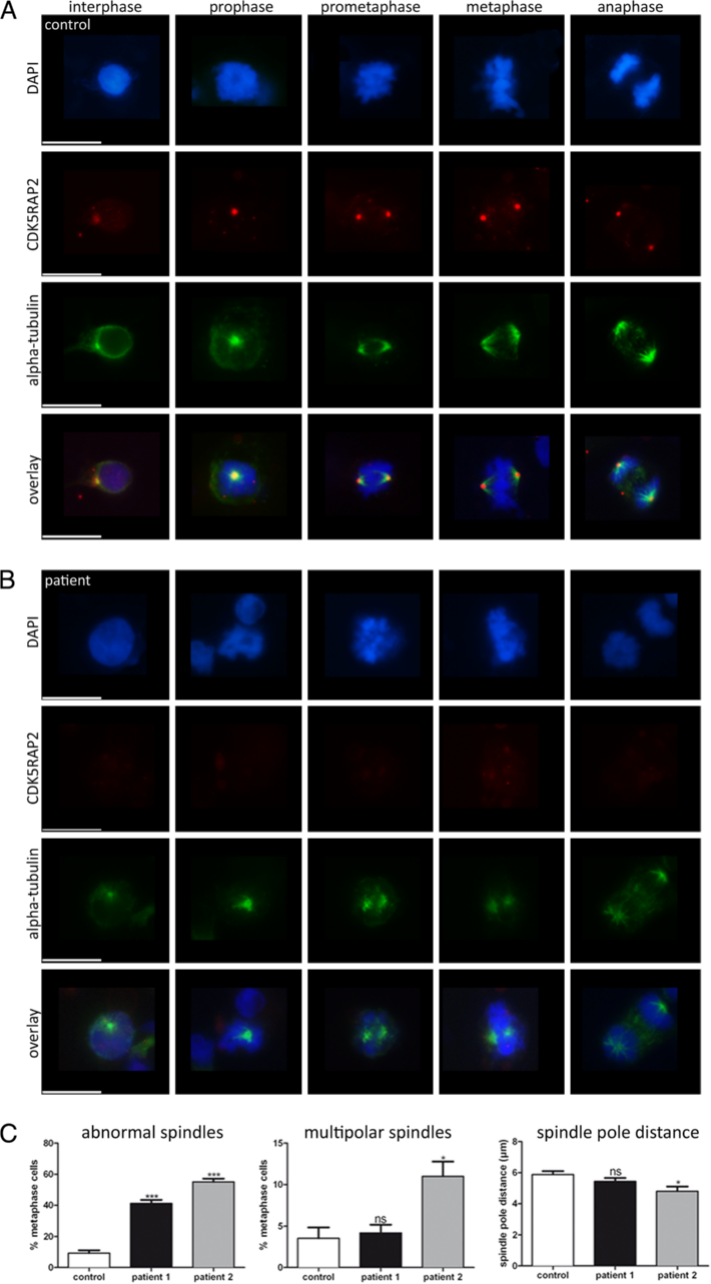
**Spindle defects in *CDK5RAP2 *mutant patients cells.** Subcellular localization of CDK5RAP2 and α-tubulin throughout the cell cycle in immortalized lymphocytes of (**A**) control and (**B**) MCPH3 patient 2. In controls, CDK5RAP2 is weak and centrosomal during interphase and shows abnormal spindle formation. In patients, spindle pole formation did not appear to be as precise as in the control cells, with the chromosomes not as uniformly positioned at the spindle poles. Cells were stained with CDK5RAP2 (red), α-tubulin (green) as a centrosomal marker, and DNA is stained with DAPI (blue). Scale bars 10 μm. (**C**) Quantification results of abnormal spindles (unfocused α-tubulin staining at spindle poles), multipolar spindles and spindle pole distance.

**Figure 7:**
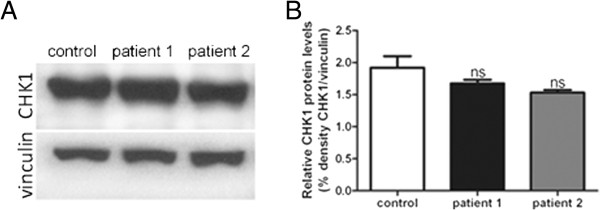
**CHK1 in *CDK5RAP2 *mutant patient and control LCLs.** (**A**, **B**) Total CHK1 protein levels detected via Western blots in immortalized lymphocytes of controls and MCPH3 patients did not differ significantly.

Because CDK5RAP2 impacts on human brain size and has been associated with progenitor proliferation, we next sought to examine the integrity of the centrosome and the establishment of the mitotic spindle apparatus in patients and controls. CDK5RAP2 colocalized with the centrosomal protein γ-tubulin throughout the cell cycle in control LCLs (Figure 3). In patient cells where CDK5RAP2 was below the detection level, we did not observe a complete loss of γ-tubulin from the centrosome nor a massive reduction of total γ-tubulin via western blot, but rather a more dispersed γ-tubulin staining around the centrosome (Figure 3). Pericentrin localization was normal in patient cells when compared to control cells (Figure 5). In addition, spindle defects with an increase of abnormal spindles with broad and unfocused poles of microtubuli (41% and 55% versus 9% of 100 counted metaphase LCLs of patient 1 and 2 versus control; One-way ANOVA, p < 0.001) were detected in *CDK5RAP2* mutant LCLs (Figure 6). There was a trend in patients towards an increase in multipolar spindles (4% and 11% versus 3.5% of 100 counted metaphase LCLs of patient 1 (not significant) and 2 (p < 0.05) versus control; One-way ANOVA) and a decrease of spindle pole distance (5.4 μm and 4.8 μm versus 5.8 μm of 100 counted metaphase LCLs of patient 1 (not significant) and 2 (p < 0.05) versus control; One-way ANOVA) in *CDK5RAP2* mutant LCLs (Figure 6). Also several LCLs from patients showed lagging chromosomes, this was significantly increased in one of the patients (patient 2) and only showed a tendency to be increased in the other patient. CHK1 protein has been shown to be downregulated in Cdk5rap2 mutant cells [14]. Although slightly reduced in both patient cell lines as compared to the control, the difference in the concentration of CHK1 protein was not significant (Figure 7).

## Discussion

In the present study, we have identified the novel nonsense mutation c.4441C > T (R1481*) in the *CDK5RAP2* gene in a homozygous constellation in two boys of Italian descent with primary microcephaly (Figures 1 and 2). We thereby, for the first time, provide detailed clinical and radiological information on MCPH3 patients of European descent. The siblings suffer from congenital microcephaly, intellectual disability, speech deficit, a tic disorder and severe behavioral problems. Further tests did not reveal any significant hearing impairment or epilepsy as a cause for the speech deficit. Therefore, although sensorineural hearing loss has been reported in two patients with mutations in *CDK5RAP2*[5,15], this is not a consistent finding in MCPH3. Both patients had microcephaly, simplified gyral pattern of the cerebral cortex, with shallow sulci anteriorly and deep sulci parietally and posteriorly, and corpus callosum hypogensis on cMRI. There was no particular evidence of reduced white matter volume in the patients, despite the fact that *CDK5RAP2* is expressed in glial cells of the developing rodent brain. Why the white matter is not more severely affected in MCPH remains unclear. Future studies will need to address the question as to what extent white matter disease also contributes to brain size reduction in MCPH patients. It is unclear whether these clinical and radiological features are also present in the previously reported three pedigrees of Pakistani descent with MCPH due to homozygous *CDK5RAP2* mutations.

In addition to the brain, we recently reported that CDK5RAP2 is widely expressed in various organs of newborn mice and human fetuses with high CDK5RAP2 mRNA and protein levels in the thymus and the kidney [7]. Moreover, it has been reported that in the MCPH3 murine model‚ “*Hertwig’s anemia”* mice display defects in multiple organs including the thymus and also have a hematopoietic phenotype (hypoproliferative anemia, leucopenia, predisposition to hematopoietic tumors) [16]. In other MCPH subtypes, individual patients have been reported with short stature (especially in MCPH1 and MCPH5 [17-19]), early puberty, renal agenesis and polycystic kidneys [17]. As this point warrants further investigation in patients, we investigated the clinical phenotype of our patients in detail with respect to multi-organ involvement. Short stature was detected in both patients up to an age of two years, but thereafter normalized in patient 1 for whom detailed data were available. This normalization of height after infancy (in contrast to the pattern of head growth) is a disease feature that has been reported similarly in patients with *ASPM* gene mutations [18]. There was no evidence of further organ involvement or malignancy, specifically no anemia or leucopenia and no kidney or thymus abnormality.

The three homozygous mutations in the *CDK5RAP2* gene reported so far, 246T > A in exon 4, 700G > T in exon 8 and 4005-15A > G in intron 26, have been proposed, but not shown, to lead to truncated proteins of 82 (Y82*), 234 (E234*) and 1334 (R1334Sfs*5) amino acids, respectively, and a loss of CDK5RAP2 function (full length protein 1893 amino acids; Figure 1). While the first and second mutant protein should lack most of the *CDK5RAP2* transcript except for the N-terminus including a part of the γTuRC-binding domain or the N-terminus including the complete γTuRC-binding domain and a part of the SMC-domain, respectively, the third protein should lack the C-terminus of CDK5RAP2, especially the c-terminal SMC domain as well as the pericentrin and the Golgi binding sites. The homozygous nonsense mutation reported here, 4441C > T in exon 30, is predicted to lead to a truncated protein of 1481 amino acids (R1481*). The resulting CDK5RAP2 protein in our patients should lack parts of the second SMC domain as well as the pericentrin and the Golgi binding sites (Figure 1). No studies on patient specimen exist that shed light on the effect of the reported *CDK5RAP2* gene mutations. We recently reported a high *CDK5RAP2* expression in proliferating progenitors of the germinal matrix and early (not mature) neurons as well as glial cells in the neocortex of murine embryos and human fetuses [7]. This is in concordance with results of neuroimaging studies in MCPH patients due to non-*CDK5RAP2* mutations demonstrating a reduced brain volume that affects especially the neocortex [17,18]. Based on results from *in vivo* and *in vitro* studies, the human MCPH phenotype is considered to be the result of a premature shift from symmetric to asymmetric neural progenitor-cell divisions (with a subsequent depletion of the progenitor pool) as well as of a reduction in cell survival [6,8].

To study the effect of the reported CDK5RAP2 gene mutation on cell proliferation in our patients, we studied EBV-transformed lymphocytes (LCLs) from both of our patients and from controls. Here, CDK5RAP2 localized to the centrosomes during each stage of the cell cycle in controls but was absent from patient cells, when assessed via immunocytology and western blots (Figure 3). The latter finding of CDK5RAP2 levels below detection levels in cells of our patients indicates that very little or no protein is present secondary to nonsense-mediated decay of the mutated transcript. In contrast to *Cdk5rap2* shRNAi studies performed on mouse tissues [20], we detected a failure of the centrosomal protein γ-tubulin to localize properly at the centrosome, while total γ-tubulin protein levels were normal in patient cells (Figure 3, Additional file 1: Figure S1). Pericentrin, which interacts with CDK5RAP2 through defined protein domains [21], was not altered in its localization in patient LCLs (Figure 5). This result is in line with those of Buchman et al. 2010 [21] who concluded from their studies in murine tissues that the centrosomal recruitment of pericentrin is not dependent upon Cdk5rap2, while the converse is true. Despite the predicted loss of the C-terminal Golgi domain in mutant CDK5RAP2, the Golgi apparatus could be visualized normally using immunostaining with GM130. However, Golgi fragmentation appeared to occur earlier during mitosis and had disappeared by prophase (Figure 4). Further, we observed an unfocused and disorganized mitotic microtubule assembly, a decrease in spindle pole distance and a trend towards more multipolar spindle poles as well as chromosome misalignment in patient cells (Figure 6). These results, although generated in lymphocytes and not neural progenitors, suggest that spindle defects and a disruption of centrosome integrity could play a role in the development of microcephaly in MCPH. On the other hand, they also underline the fact that, at least in the cells studied, despite a lack of normal CDK5RAP2, a centrosomal structure can still be formed, microtubuli can still be nucleated to the centrosomes and cells can still divide. Since microcephalin and pericentrin regulate mitotic entry via centrosome-associated Chk1 [22] and Chk1-downregulation has been demonstrated in mutant Cdk5rap2 cells [14], we analyzed CHK1 levels in CDK5RAP2 mutant and control cells. Although slightly reduced in both patients, there was not a significant decrease in total CHK1 levels in patients cells (Figure 7).

Brain size at birth is largely determined by the relative rates of proliferation and cell death. By highlighting the clinical, radiological and cellular phenotype of MCPH3 patients, we offer a further glimpse into how a disruption of the *CDK5RAP2* gene may impact on the development in humans. Further analysis of patient samples provides a means to investigate processes that cause MCPH and to verify mechanisms described in other model systems and in settings where animal models are neither sufficient nor satisfactory.

## Competing interests

The authors declare no competing interests in the preparation or publication of the data in this manuscript.

## Authors’ contributions

AM and DMR were responsible for the project conception and wrote the manuscript. LI, NK and ON performed the lymphocyte analysis, generated figures and proofread the manuscript. KM and KS performed genetic analysis and compiled clinical data. HR and MB attended the patients and provided clinical data. All authors read and approved the final manuscript.

## Supplementary Material

Additional file 1: Figure S1 Gamma tubulin in *CDK5RAP2* mutant patient and control LCLs. (A, B) Total gamma tubulin protein levels detected via Western blots in immortalized lymphocytes of controls and MCPH3 patients did not differ significantly. Click here for file
