# Supplementary figures and images for: Clinical and cellular features in patients with primary autosomal recessive microcephaly and a novel CDK5RAP2 mutation
Source: Orphanet J Rare Dis. 2013 Apr 15;8:59. doi: 10.1186/1750-1172-8-59 (PMC3639195; doi:10.1186/1750-1172-8-59)

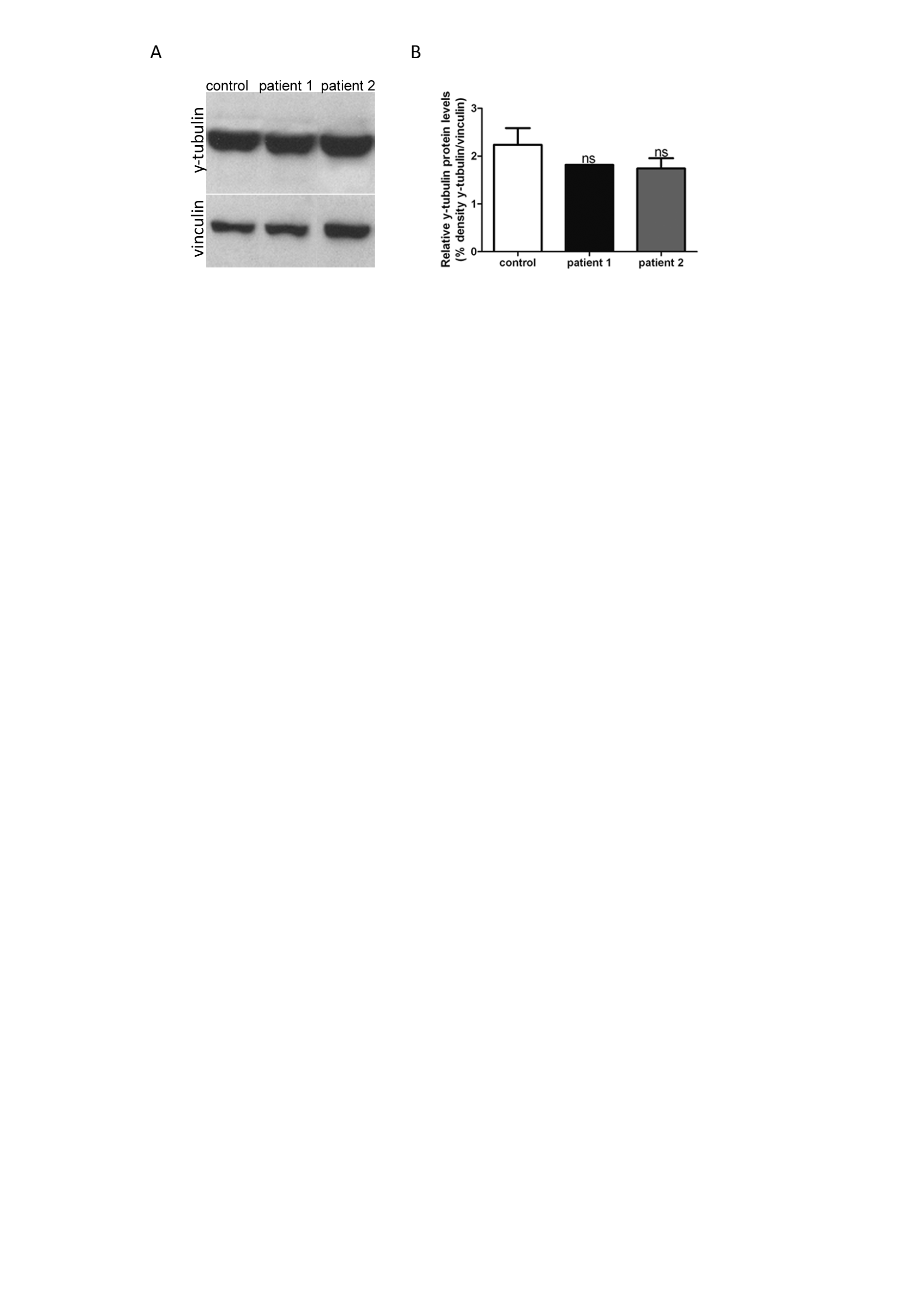

Supplement: Additional file 1: Figure S1 — Gamma tubulin in CDK5RAP2 mutant patient and control LCLs. (A, B) Total gamma tubulin protein levels detected via Western blots in immortalized lymphocytes of controls and MCPH3 patients did not differ significantly. [file 1750-1172-8-59-S1.tiff]
